# Sjögren’s syndrome and Neuromyelitis Optica spectrum disorders (NMOSD) – a case report and review of literature

**DOI:** 10.1186/s12883-014-0200-5

**Published:** 2014-10-09

**Authors:** Apoorva Jayarangaiah, Rahul Sehgal, Narendranath Epperla

**Affiliations:** Department of Internal Medicine, Marshfield Clinic, Marshfield, WI 54449 USA; Department of Rheumatology, Marshfield Clinic, Marshfield, WI 54449 USA; Department of Clinical Research, Marshfield Clinic Research Foundation, Marshfield, WI 54449 USA

**Keywords:** Neuromyelitis optica, Neuromyelitis optica spectrum disorders, Sjogren’s syndrome, Aquaporin 4 antibody

## Abstract

**Background:**

Neuromyelitis optica (NMO) is a rare relapsing auto-immune disease of the central nervous system which is sometimes found in association with other autoimmune disorders including Sjogren’s syndrome. We present the case of a middle aged female with Sjogren’s syndrome (SS) and Neuromyelitis optica spectrum disorders (NMOSD) who had a rapidly declining neurological illness that responded to immunosuppressive therapy.

**Case presentation:**

A 51-year-old female with Sjogren’s syndrome and recent history of varicella zoster infection presented with right upper and lower extremity weakness of one week duration. She was noted to have contrast enhancement at C2-C4 cord levels on cervico-thoracic MRI. Comprehensive work up was negative except for presence of a mild lymphocytic pleocytosis and oligoclonal bands in the CSF. She was diagnosed with transverse myelitis secondary to varicella zoster infection and was treated with high dose steroids in addition to acyclovir with improvement in her symptoms. Two months later she developed left upper and lower extremity weakness, bilateral dysesthesias and urinary incontinence. Repeat MRI of the cervico-thoracic spine revealed worsening enhancement at lower cervical cord levels (C5-7) with extension to T1. CSF analysis was unchanged; however immunological work up was abnormal for elevated NMO-IgG/AQP4 antibody. She was diagnosed with NMOSD and was treated with immunosuppressive therapy. Initially with IV methylprednisone and Cyclophosphamide therapy followed by Mycophenolate mofetil (MMF) maintenance therapy with good response. Repeat MRI 6 months later showed near complete resolution of previous abnormal cord signal changes.

**Conclusion:**

One needs to recognize the relationship between autoimmune diseases especially SS and NMOSD. The presence of NMO antibody has been associated with a relapsing disease course and a careful follow-up, besides use of remission maintenance agents such as MMF and Azathioprine are important to consider.

## Background

Sjögren’s syndrome (SS) is a chronic systemic disease characterized by inflammation and dysfunction of exocrine glands. Up to 65% of primary SS patients can experience extra glandular features including pulmonary, gastrointestinal, hematologic and neurologic disorders [1]. Neurologic disorders are severe extraglandular features of SS. Longitudinally extensive myelitis has been reported in SS [2,3]. Neuromyelitis optica (NMO) also known as Devic syndrome is a rare relapsing auto-immune disease of the central nervous system (CNS) which is sometimes found in association with other autoimmune disorders, including SS. Based on the revised criteria by Wingerchuck et al, a diagnosis of NMO can be made in the presence of both absolute and two of three supportive criteria [4]. The absolute criteria include optic neuritis and myelitis; while the supportive criteria are magnetic resonance imaging (MRI) evidence of a contiguous spinal cord lesion (3 or more segments in length), MRI brain non-diagnostic for multiple sclerosis and serological evidence of NMO-IgG or aquaporin 4 (AQP4) antibodies. NMO spectrum disorders (NMOSD) includes a wide range of neurologic conditions that express NMO antibody and share features with NMO but do not meet the strict diagnostic criteria specified previously [5].

Herein we present the case of a middle aged female with SS and NMOSD who had a rapidly declining neurological illness that responded to immunosuppressive therapy. Prompt recognition and treatment can alter the course of this uncommon yet devastating illness, though the prognosis and response to therapy is not always favorable.

## Case presentation

A 51 year old right hand dominant Caucasian female with history of hypothyroidism (microsomal and thyroid antibody positive), celiac disease and SS (seropositive for SSA, ANA, hypergammaglobulinemia, intermittent parotid swelling with mild oral/ocular sicca) experienced right C5 dermatomal varicella zoster infection seven weeks previously and then presented with right upper and right lower extremity weakness of one week duration. The symptoms got progressively worse to the point that she had difficulty with ambulation. She had associated burning dysesthesias of left thigh. The patient’s neurological examination was remarkable for hypertonia, decreased power (3/5), hyperreflexia along with sensory loss in the right upper and lower extremity and hyperesthesia in the entire left lower extremity.

**Figure 1:**
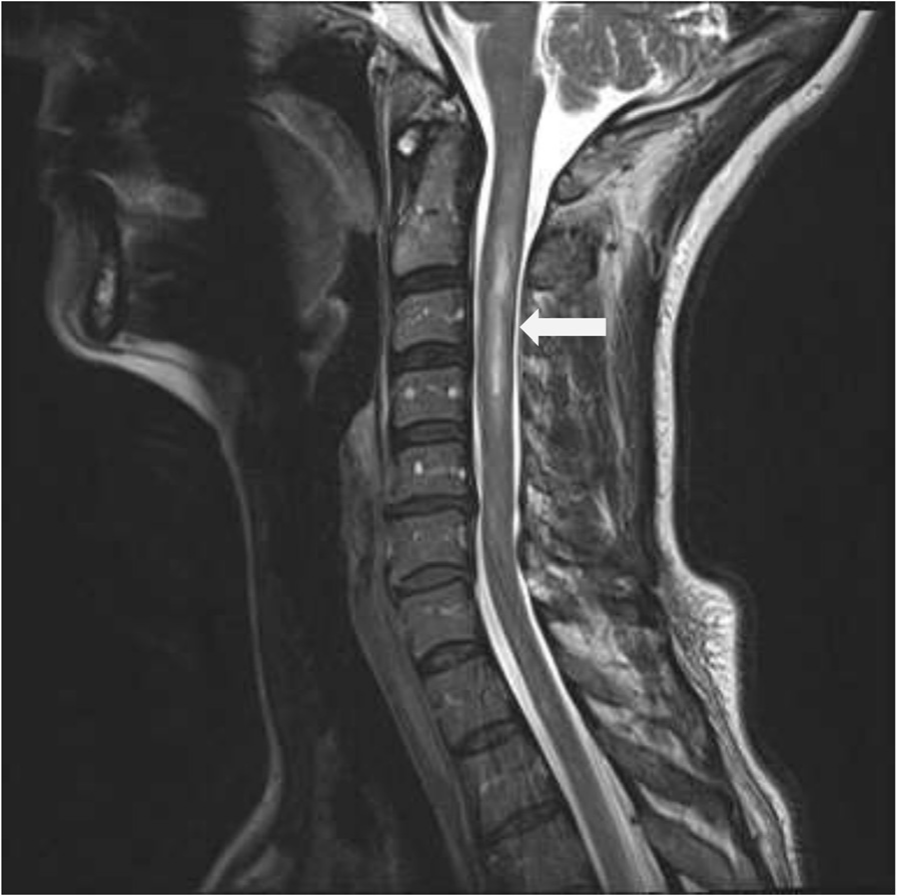
**MRI T2 weighted sequence of the cervical region showing contrast enhancing lesion from C2-C4.**

Gadolinium contrast enhanced magnetic resonance imaging (MRI) of the head was normal but MRI of the cervico-thoracic spine revealed an enhancing intramedullary lesion from C2 to C4, centrally into the right of the midline with signal changes at the T1 level without enhancement or expansive appearance (Figure 1). Cerebrospinal fluid (CSF) analysis was abnormal for a mild lymphocytic pleocytosis, marked elevation of IgG and prominent oligoclonal bands (Table 1). Comprehensive infectious workup including blood cultures for bacteria, polymerase chain reaction for varicella zoster, herpes simplex virus, and serologies for Lyme disease and syphilis were negative. Differentials for non-infectious inflammatory myelitis were considered; including paraneoplastic myelitis and myelitis with Sjogren’s syndrome. NMO antibodies were not obtained during this presentation. The patient was treated with 1 gram of intravenous (IV) methylprednisone and 800 mg of oral acyclovir for 5 days for presumptive diagnosis of transverse myelitis secondary to varicella zoster infection with improvement in her symptoms. She was discharged on oral prednisone with instructions to taper and discontinue the drug over the next 1 week.

**Table 1:** **Comparison of CSF analysis during two separate hospitalizations**

***Tests***	***Initial Sample***	***Subsequent Sample***	***Normal***
Total nucleated cells	11	16	0-5
RBC	1245	38	0
Neutrophils	15	01	0-6
Lymphocytes	74	82	40-80
Protein	60	44	15-45
Gross appearance	Colorless	Colorless	Colorless
Oligoclonal bands	Present	Present	
IgG Index	15.8	10.6	0-6.6
IgG/Albumin ratio	0.43	0.47	0-0.27
Culture (aerobic, fungal, viral)	Negative	Negative	Negative
Cytology	Negative	Negative	Negative
PCR (Varicella, Lyme)	Negative	Negative	Negative

**Figure 2:**
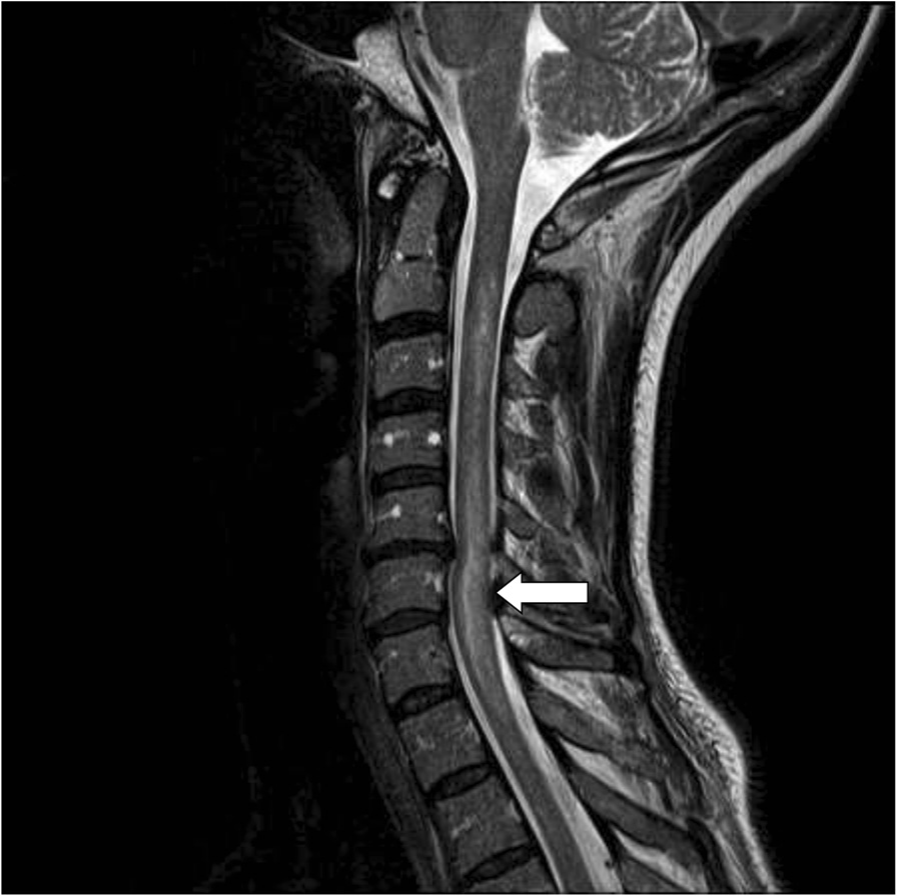
**MRI T2 weighted sequence of the cervical MRI showing abnormal signal changes extending from C2-T1 with worsening enhancement at lower cervical cord levels.**

Two months later, she developed left upper and lower extremity weakness, bilateral lower extremity dysesthesias and urinary incontinence. Examination confirmed 4/5 left hemiparesis with brisk reflexes, bilateral ankle clonus along with pinprick loss in both lower extremities. Repeat MRI of the spine revealed worsening enhancement at lower cervical cord region (C5-7) with extension to T1 level (Figure 2). Repeat CSF analysis showed a lymphocytic pleocytosis, increased IgG synthesis index and positive oligoclonal bands (Table 1). Immunological work up was abnormal for NMO-IgG/AQP4 antibody (Table 2). An ophthalmologic evaluation was negative for optic neuritis. The patient’s relapsing neurological illness was felt to represent NMOSD from underlying long standing SS. She was treated with 1 gram of IV methylprednisone and started on 1 gram of IV cyclophosphamide (CTX) therapy for worsening motor-sensory deficit. She was discharged on high dose oral prednisone and continuation of monthly CTX infusions.

**Table 2:** **Summary of the immunological work up**

***Immunology***	***Patient***	***Normal***
ANA	*>1:640 (speckled pattern)*	<1:40
Anti-ds DNA	<1:10	<1:10
SS-A/Ro antibodies	*>8.0*	0.0-0.9
SS-B/Lo antibodies	<0.2	0.0-0.9
Scl-70 antibodies	<0.2	0.0-0.9
Jo 1 antibodies	0.2	0.0-0.9
NMO-IgG (AQP4 antibody)	*>160*	<1.6
Anti CCP antibodies	4.0	0.0-19.9
dRVVT	34.5	32.4-43.5
PTT-LA	39	42-64
Anticardiolipin screen	Negative	Negative
Beta 2 Glycoprotein 1 screen	Negative	Negative

**Figure 3:**
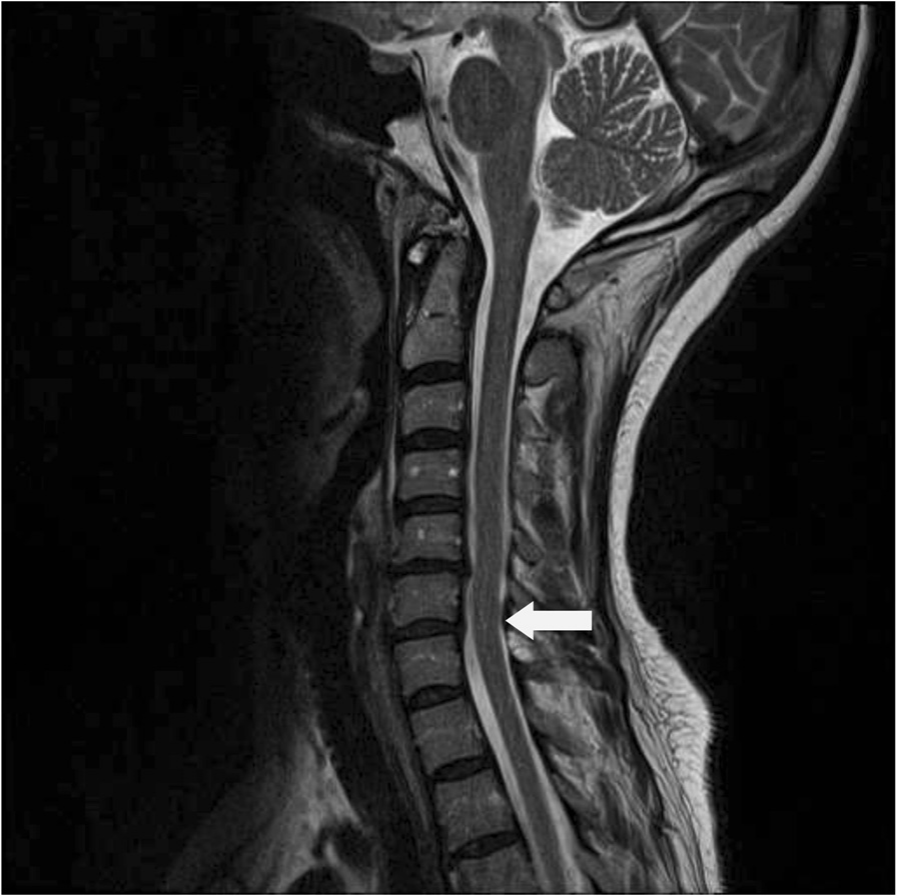
**MRI T2 weighted sequence of the cervical MRI after Prednisone, Cyclophosphamide and Mycophenolate therapy, demonstrates improvement and near resolution of previously noted signal abnormalities.**

After her second monthly CTX infusion, patient had a relapse with right upper and lower extremity weakness, dysesthesias in the parieto-occipital scalp region and right leg along with urinary incontinence. The patient was placed on 5 days of pulse IV steroids with improvement in her symptoms. She was discharged on 60 mg of daily prednisone which was slowly tapered over 6 months. She continued to show clinical improvement despite persistence of NMO-IgG antibodies. After 5 infusions of monthly CTX therapy, maintenance treatment with Mycophenolate Mofetil (MMF) was started. She has remained on MMF without recurrence of symptoms for over 18 months. She has no residual motor or sensory deficits. Repeat MRI of the cervico-thoracic spine 6 months after her last CTX infusion, prednisone and MMF showed near complete resolution of previous abnormal cord signal changes (Figure 3).

## Discussion

A wide spectrum of neurologic manifestations may accompany primary SS. These can include asymptomatic brain lesions on MRI [6] to symptomatic brain lesions, meningitis, myelopathy, cranial neuropathy, sensorimotor polyneuropathy and mononeuritis multiplex. Spinal cord involvement in primary SS may resemble primary progressive multiple sclerosis, progressive myelopathy or transverse myelitis. The pathogenesis of CNS involvement in primary SS is unclear. A vasculitic process and autoimmune demyelination have been proposed to be possible mechanisms for this process [7,8]. Unlike systemic lupus erythematosus (SLE), patients with primary SS do not demonstrate anti-neuronal or anti-ribosomal antibodies to suggest additional immune-pathologic mechanism other than humoral immune response [9]. Primary SS can mimic as well as coexist with multiple sclerosis [10] though only a very small percentage of multiple sclerosis patients have detectable SS [11]. Nonetheless it can pose a significant diagnostic and therapeutic challenge in clinical practice.

The differential diagnosis for neurologic manifestations of SS is broad including infections, neoplasms and metabolic disorders; work-up in an individual patient must be thorough to exclude them. Tests for anti-phospholipid antibodies, CSF exam and imaging with MRI or single photon emission computed tomography scans must be considered when entertaining a diagnosis of neurologic SS. Brain biopsy is done only in rare instances when the work up is unrevealing and precise etiology is unclear.

NMOSD is a relapsing neurological illness that has been sometimes reported in association with primary SS and SLE. NMO is characterized by recurrent episodes of myelitis and optic neuritis and most patients have a unique antibody against NMO IgG/ AQP4. Patients who have NMO IgG/AQP4 antibodies have frequent relapses compared to those who do not have the antibodies [12].

NMOSD is manifested by loss of AQP4, which is a bidirectional water channel found in the cell membrane of foot processes of the astrocytes and on the ependymal cells in the CNS [13]. The presence of NMO IgG antibodies against these AQP4 channels aids in the diagnosis of NMO in patients with neurologic manifestations [14,15]. Following the discovery of NMO IgG antibodies, Weinshenker et al reported the specificity of NMO IgG antibodies to be 91% and the sensitivity to be 75%, in distinguishing between MS and NMO [16]. Various HLA alleles such as HLA-DRB1*03, HLA-DPB1*0501 and structural changes in the AQP4 channel have been affiliated with an increased susceptibility to developing NMO [17]. Studies have shown that an inflammatory process such as an infection (viral or bacterial) can be a trigger in developing immunogenicity [18]. It may also play a role in damaging the blood brain barrier allowing NMO IgG penetration [19]. NMO IgG antibodies cause destruction of the AQP4 channels on the astrocyte foot processes by complement mediated cytotoxicity via the lytic complex C5b9 [20]. Antibody production by B-cells and T-helper cell activation of cytokines such as IL-16 and IL-17 are thought to play a crucial role in the subsequent destruction of the AQP4 channels [21,22]. The consequential loss of oligodendrocytes results in demyelination leading to the neurological signs seen in NMO [23]. The lesions in NMO are characterized by extensive infiltration, acute axonal loss and fibrosis [24].

There is not enough evidence yet to consolidate the etiologic relationship between infectious or parainfectious NMO syndromes; this relationship currently only exists based on a temporal spectrum [25]. A review of parainfectious NMO syndromes by Sellner et al demonstrated 6 out of 11 patients with prior VZV infection (with vesicular eruptions) who presented with features of transverse myelitis and visual problems [25]. A proportion of the patients fit the criteria for NMO spectrum; however most cases were not associated with recurrent attacks and all but one were seronegative for AQ4 antibodies [25]. A case report by Heerlein et al demonstrates a similar patient as ours who presented with features of LETM within two weeks following herpes zoster rash. The patient was seropositive for AQP4 antibodies, with subsequent remission of symptoms and disappearance of AQP4 antibodies following plasmapheresis [26].

Relapsing NMO carries a poor prognosis, thereby validating the need for early testing and treatment. It has been reported to have a 5 year survival rate of approximately 80% [27]. The cumulative effect of recurrent attacks renders a debilitating outcome, including permanent disabilities requiring assistive devices for ambulation and monocular blindness [27]. A proportion of deaths occurring within the first 5 years are due to respiratory failure [27].

NMO and NMOSD are rare diseases with no consensus on treatment guidelines. The treatment options are empiric and based on disease severity ranging from plasmapheresis, corticosteroids, CTX and rituximab to oral agents such as azathioprine, MMF and methotrexate. Early initiation of systemic steroids alone or in combination with other immunosuppressive therapies is most often used. In our patient with relapsing disease course, combination of steroids and CTX was successful in achieving disease stabilization, resolution of cord enhancing lesions and recovery from a declining clinical course. MMF has helped maintain disease remission despite persistent NMO antibody detectable in the serum.

The rationale for therapy with MMF can be explained by the predominant role of humoral immunity in NMO. A subgroup analysis in a small retrospective trial in patients receiving a median dose of 2 gm/day of MMF demonstrated a decrease in the median annual relapse rate from 1.3 (range 0.23-11.8) in the pretreatment group to 0.09 (range 0-1.5) in the post treatment group at a median follow up of 28 months [28]. A single case report published in 2006 reported a 9 y/o girl treated with MMF successfully with no relapse at 2 years following development of vertebral fractures with steroids and continued relapses on Azathioprine [29]. Rituximab has similarly shown to reduce the levels of NMO IgG/ AQP4 [30,31] and thereby clinical improvement.

## Conclusion

The spectrum of neurologic disorders in Sjögren’s syndrome remains broad and diagnosis is frequently delayed. Conventional treatment strategies using corticosteroids are effective for acute flare ups of severe manifestations such as NMO, NMOSD and transverse myelitis. Treatment with cytotoxic agents and rituximab are useful in achieving disease remission, although treatment intensity should be guided by severity of clinical presentation. The presence of NMO antibody has been associated with a relapsing disease course and a careful follow-up, besides use of remission maintenance agents such as MMF and Azathioprine are important to consider. There remain several unanswered questions including duration of maintenance medications and utility of following NMO titers in predicting future risks of flare. Further studies with long term patient follow-ups will answer these potentially important clinical questions.

### Consent

Written informed consent was obtained from the patient for publication of this case report and any accompanying images. A copy of the written consent is available for review by the Editor-in-Chief of this journal.

## Abbreviations

NMO: Neuromyelitis optica
NMOSD: Neuromyelitis optica spectrum disorders
MMF: Mycophenolate mofetil
MRI: Magnetic resonance imaging
